# Clinical and prognostic differences in mild to moderate AECOPD with and without emphysema: a 3-year multicenter prospective study

**DOI:** 10.3389/fmed.2026.1853642

**Published:** 2026-06-24

**Authors:** Jiaqi Pu, Mingjing Yu, Hailong Wei, Huiqing Ge, Huiguo Liu, Jianchu Zhang, Pinhua Pan, XiuFang Xie, Mengqiu Yi, Xianhua Li, Lina Cheng, Hui Zhou, Jiarui Zhang, Jiaxin Zeng, Xueqing Chen, Haixia Zhou, Qun Yi

**Affiliations:** 1 Department of Respiratory and Critical Care Medicine, West China Hospital, Sichuan University, Chengdu, Sichuan, China; 2 State Key Laboratory of Respiratory Health and Multimorbidity, West China Hospital, Sichuan University, Chengdu, China; 3 Department of Respiratory and Critical Care Medicine, People's Hospital of Leshan, Leshan, Sichuan, China; 4 Department of Respiratory and Critical Care Medicine, Sir Run Run Shaw Hospital, Zhejiang University School of Medicine, Hangzhou, Zhejiang, China; 5 Department of Respiratory and Critical Care Medicine, Tongji Hospital, Tongji Medical College, Huazhong University of Science and Technology, Wuhan, Hubei, China; 6 Department of Respiratory and Critical Care Medicine, Union Hospital, Tongji Medical College, Huazhong University of Science and Technology, Wuhan, Hubei, China; 7 Department of Respiratory and Critical Care Medicine, Xiangya Hospital, Central South University, Changsha, Hunan, China; 8 Department of Respiratory and Critical Care Medicine, The First People’s Hospital of Neijiang City, Neijiang, Sichuan, China; 9 Department of Emergency, First People's Hospital of Jiujiang, Jiujiang, Jiangxi, China; 10 Department of Respiratory and Critical Care Medicine, The Affiliated Hospital of Chengdu University, Chengdu, Sichuan, China; 11 Sichuan Cancer Hospital, University of Electronic Science and Technology of China, Chengdu, Sichuan, China

**Keywords:** computed tomography, COPD exacerbation, emphysema phenotype, mortality, prognosis

## Abstract

**Introduction:**

The prognostic significance of the computed tomography (CT)-defined emphysema phenotype in patients hospitalized with mild-to-moderate acute exacerbation of chronic obstructive pulmonary disease (AECOPD) remains poorly defined.

**Materials and methods:**

This prospective multicenter cohort study analyzed data from the MAGNET AECOPD Registry. We included patients hospitalized with mild-to-moderate AECOPD (Rome criteria) who underwent chest CT within 24 h of admission. Participants were categorized into emphysema and non-emphysema groups based on visual CT assessment. The primary outcome was 3-year all-cause mortality; secondary outcomes included rehospitalization and in-hospital events. Propensity score matching balanced baseline characteristics.

**Results:**

Among 5,713 eligible patients, 4,558 (79.8%) had emphysema. After 1:1 propensity score matching (*n* = 2,258), the emphysema group demonstrated more severe acute disease, with higher proportions of respiratory failure (12.1% vs. 3.4%, *p* < 0.001), and elevated inflammatory markers (all *p* < 0.001). Interestingly, the emphysema group exhibited significantly lower 3-year all-cause mortality (24.8% vs. 30.2%, log-rank *p* = 0.048) despite higher rates of non-invasive ventilation requirement (24.1% vs. 19.1%, *p* = 0.005) and longer hospital stays (11 vs. 10 days, *p* < 0.001). The non-emphysema group carried a heavier burden of cardiovascular comorbidities including hypertension (41.5% vs. 32.7%, *p* < 0.001) and heart failure (18.7% vs. 13.6%, *p* = 0.001).

**Conclusion:**

In mild-to-moderate AECOPD, the emphysema phenotype predicts higher acute morbidity yet lower long-term mortality. This finding suggests potential distinct pathophysiological pathways and supports further investigation of CT-based phenotyping for personalized management in COPD.

**Clinical trial registration:**

http://www.chictr.org.cn/showproj.aspx?proj=121626, Identifier ChiCTR2100044625.

## Highlights

1. This is the first large-scale study focusing exclusively on mild-to-moderate AECOPD patients, suggesting a potential dissociation between short-term morbidity and long-term mortality based on CT phenotype.
2. The emphysema phenotype is linked to more severe acute disease (higher inflammation, more respiratory failure) yet lower 3-year all-cause mortality (*p* = 0.048).
3. The elevated mortality in non-emphysematous patients is potentially driven by a heavier burden of cardiovascular comorbidities like hypertension and heart failure.
4. Our hypothesis-generating findings suggest a personalized management strategy: comorbidity-focused care for non-emphysema and optimized respiratory support for emphysema phenotypes, pending confirmation in future studies.

## Introduction

Chronic Obstructive Pulmonary Disease (COPD) stands as a leading global cause of morbidity and mortality, representing a substantial and escalating public health burden (1, 2). The disease is defined by persistent respiratory symptoms and progressive airflow limitation, stemming from a combination of small airway disease and parenchymal destruction, or emphysema (3). This pathophysiological diversity drives the emergence of distinct clinical phenotypes with profound differences in clinical outcomes (4, 5), among which the emphysema phenotype, readily identified on computed tomography, is consistently associated with more severe functional impairment and a unique profile of disability (6). Large-scale cohort studies have further validated the prevalence and clinical relevance of such phenotypic stratification across diverse populations (7).

Central to these pathological processes is emphysema, which constitutes not merely a component but a central pathological substrate of COPD, critically influencing disease heterogeneity. A growing body of evidence delineates the emphysema-predominant phenotype as a distinct clinical entity, characterised by accelerated functional decline and a heightened symptom burden, in contrast to non-emphysematous forms which often present with a higher prevalence of systemic comorbidities (8). Importantly, even in mild to moderate disease, the presence of emphysema has been linked to increased exacerbation frequency and a more rapid deterioration in health status, underscoring its prognostic significance (9). Despite these insights, a critical gap remains in our understanding of how emphysema in the early stages of COPD influences the long-term trajectory, particularly regarding hard endpoints such as mortality. Crucially, acute exacerbations of COPD (AECOPD) are pivotal events that not only reflect underlying disease severity and phenotype but also independently drive disease progression and long-term outcomes. Determining whether early computed tomography-based identification of emphysema can effectively stratify future risk at the time of a mild-to-moderate exacerbation is therefore a pressing clinical research priority.

To address this unmet need, we undertook a prospective, multicentre study to systematically evaluate differences in baseline characteristics and long-term prognosis, including exacerbation risk and mortality, between patients hospitalized with mild to moderate AECOPD with and without emphysema. We hypothesised that the emphysema phenotype confers a unique risk profile from the earliest disease stages. Our findings aim to refine the early phenotypic stratification of AECOPD, thereby informing the development of targeted management strategies for high-risk subgroups at the early disease stage, a critical window for intervention.

## Methods

### Study design and population

This prospective cohort study analyzed data from the MAGNET AECOPD Registry (ChiCTR2100044625), which has been described in detail previously (10). The present analysis represents a secondary analysis of this registry data. Briefly, the registry consecutively enrolled patients hospitalized for AECOPD across ten tertiary centers in China between September 2017 and July 2021. For the present analysis, we identified a subpopulation of patients experiencing a mild-to-moderate AECOPD event, as defined by the Rome classification (11), who had undergone computed tomography (CT) within 24 h of admission. The study protocol was approved by the central ethics committee at West China Hospital, Sichuan University, and all participating centers. Written informed consent was obtained from all subjects.

### Diagnostic and severity classification criteria

All included patients had AECOPD as the primary admission diagnosis. AECOPD was defined as an acute worsening of respiratory symptoms (e.g., dyspnea, cough, or sputum purulence) that necessitated a change in medication in a patient with spirometrically confirmed COPD (post-bronchodilator forced expiratory volume in one second [FEV₁]/forced vital capacity [FVC] < 0.70). Exacerbation severity was assessed using the Rome classification (11), and only events categorized as mild or moderate were included in this study. The validity and reliability of this classification system have been established in the previous cohort (12) and also within our study population (13).

### CT-based phenotyping and group stratification

The study cohort was stratified into two groups based on the presence or absence of visually detected emphysema on admission standard chest CT scans. The images were independently reviewed by two board-certified thoracic radiologists, each with over ten years of experience, who were blinded to all clinical data. Emphysema was qualitatively defined as the presence of visually apparent low-attenuation areas, vascular pruning, or bullae, in accordance with the Fleischner Society criteria (14). Inter-observer agreement was substantial (Cohen’s kappa = 0.82). The few discrepant cases were resolved through a consensus reading with a third senior investigator.

### Data collection and follow up

Clinical data were prospectively collected using a standardized case report form by extracting electronic medical records within 24 h of admission. The collected variables included baseline characteristics, comorbidities, clinical manifestations, laboratory tests, imaging findings, in-hospital treatments, and short-term and long-term clinical outcomes. The primary endpoint was 3-year all-cause mortality. Secondary endpoints included rehospitalization for COPD acute exacerbation, in-hospital mortality, requirement for mechanical ventilation (invasive or non-invasive), intensive care unit (ICU) admission, length of hospital stay (LOS), and administration of systemic corticosteroids or antibiotics. To ensure complete endpoint ascertainment, a structured follow-up procedure was conducted every six months for up to 3 years post-discharge at West China Hospital of Sichuan University. This included telephone interviews, clinical visit reviews, and electronic medical record audits, thereby maximizing data completeness for mortality outcomes.

### Statistical analysis

Continuous variables were compared using independent samples t-tests or Mann–Whitney U tests, and are presented as mean ± standard deviation or median with interquartile range (IQR). Categorical variables were compared using the chi-square test and are expressed as percentages (*n*, %). We compared baseline characteristics, comorbidities, in-hospital complications, clinical manifestations, laboratory tests, and clinical outcomes between AECOPD patients with and without emphysema. To control for potential confounding factors, 1:1 propensity score matching (PSM) was performed using nearest-neighbor matching with a caliper width of 0.2 standard deviations. Matching covariates included age, sex, and smoking status. Notably, baseline lung function was not incorporated as a matching covariate due to substantial missingness; however, as presented in Table 1, the available data indicated that the distributions of FEV₁% predicted and FEV₁/FVC ratio were comparable between patients with and without emphysema, both before and after matching. After matching, all standardized mean differences (SMD) were below 0.1, indicating good balance between the groups. Survival differences were evaluated using Kaplan–Meier analysis with log-rank tests to compare AECOPD patients with and without emphysema. Additionally, survival curves were plotted separately for AECOPD patients with mild and moderate Rome severity classifications. To describe the study population, crude 3-year all-cause mortality proportions were calculated as the number of deaths divided by the number of patients who completed the 3-year follow-up period (subjects lost to follow-up were excluded from the denominator). For survival analysis, Kaplan–Meier curves were generated to visualize survival differences between groups, and patients who were lost to follow-up or withdrew before the 3-year endpoint were censored at the time of their last known contact. Multivariable Cox proportional hazards regression analysis was performed to identify independent predictors of 3-year all-cause mortality. Covariates were selected based on previously reported prognostic factors in AECOPD literature, including age, sex, pneumonia, heart failure, cor pulmonale, and diastolic blood pressure (15). All statistical analyses were performed using SPSS 26 and R 4.2.3, with a two-tailed significance level of *p* < 0.05.

**Table 1:** Baseline characteristics of AECOPD patients with vs. without emphysema before and after propensity score matching.

Variables	Before PSM			After 1:1 PSM		
	With emphysema	without emphysema	*p*-value	With emphysema	Without emphysema	*p*-value
Number of patients	4,558	1,155	/	1,129	1,129	/
Baseline characteristics						
Gender (female), %	942 (20.7)	367 (31.8)	**<0.001**	349 (30.9)	355 (31.4)	0.819
Age (years) (mean ± SD)	72.63 ± 10.25	71.01 ± 11.47	**<0.001**	71.44 ± 10.57	71.55 ± 10.74	0.817
BMI, kg/m^2^ (mean ± SD)	21.43 ± 3.93	22.81 ± 4.22	**<0.001**	21.57 ± 3.86	22.82 ± 4.22	**<0.001**
Current smoker, %	908 (19.9)	165 (14.3)	**<0.001**	157 (13.9)	159 (14.1)	0.951
Long-term oxygen therapy, %	206 (17.8)	26 (14.1)	0.250	41 (23.3)	25 (14.3)	**0.037**
Frequency of exacerbations (≥2 times/year), %	575 (32.0)	121 (25.7)	**0.029**	79 (18.8)	120 (26.0)	**<0.001**
FEV1/FVC	51.89 ± 14.06	51.35 ± 13.04	0.839	51.82 ± 14.27	52.35 ± 14.68	0.866
FEV1/pred	57.90 ± 23.09	51.07 ± 22.98	0.127	57.44 ± 23.29	52.25 ± 23.25	0.310
Comorbidities						
Hypertension, %	1,582 (34.7)	473 (41.0)	**<0.001**	369 (32.7)	468 (41.5)	**<0.001**
Heart failure, %	639 (14.0)	213 (18.4)	**<0.001**	153 (13.6)	211 (18.7)	**0.001**
Pulmonary hypertension, %	153 (3.4)	44 (3.8)	0.468	32 (2.8)	42 (3.7)	0.300
OSAHS	20 (0.4)	18 (1.6)	**<0.001**	4 (0.4)	18 (1.6)	**0.004**
Cor pulmonale, %	1,211 (26.6)	220 (19.0)	**<0.001**	427 (37.8)	214 (19.0)	**<0.001**
Connective tissue disease, %	65 (1.4)	28 (2.4)	**0.020**	21 (1.9)	27 (2.4)	0.465
Diabetes, %	687 (15.1)	206 (17.8)	**0.025**	205 (18.2)	204 (18.1)	1.000
Osteoporosis, %	150 (3.3)	52 (4.5)	**0.049**	57 (5.0)	52 (4.6)	0.700
Parkinson’s disease, %	47 (1.0)	11 (1.0)	0.870	11 (1.0)	11 (1.0)	1.000
Chronic renal insufficiency, %	210 (4.6)	61 (5.3)	0.347	47 (4.2)	61 (5.4)	0.198
Gastroesophageal reflux, %	64 (1.4)	20 (1.7)	0.406	16 (1.4)	20 (1.8)	0.619
Anxiety or depression, %	53 (1.16)	13 (1.13)	1.000	10 (0.9)	13 (1.2)	0.681
In-hospital complications						
Rome classification, %			<0.001			<0.001
Mild	2,212 (48.5)	655 (56.7)		519 (46.0)	639 (56.6)	
Moderate	2,346 (51.5)	500 (43.3)		610 (54.0)	490 (43.4)	
Pneumonia, %	1,310 (28.7)	316 (27.4)	0.357	376 (33.3)	307 (27.2)	**0.002**
Respiratory failure, %	466 (10.2)	41 (3.5)	**<0.001**	137 (12.1)	38 (3.4)	**<0.001**
Sepsis, %	28 (0.6)	5 (0.4)	0.522	9 (0.8)	5 (0.4)	0.430
Clinical manifestations						
Cough, %	4,300 (94.3)	1,055 (91.3)	**<0.001**	1,067 (94.5)	1,032 (91.4)	**0.005**
Purulent sputum, %	877 (19.2)	192 (16.6)	**0.044**	363 (32.2)	185 (16.4)	**<0.001**
Fever, %	628 (13.8)	167 (14.5)	0.569	183 (16.2)	165 (14.6)	0.317
Chest pain, %	286 (6.3)	78 (6.8)	0.595	75 (6.6)	76 (6.7)	1.000
Hemoptysis, %	158 (3.5)	41 (3.5)	0.928	37 (3.3)	39 (3.5)	0.906
Systolic blood pressure (mmHg, mean ± SD)	131.99 ± 19.53	130.24 ± 19.92	**0.008**	130.39 ± 19.81	130.38 ± 19.95	0.988
Diastolic blood pressure (mmHg, mean ± SD)	78.64 ± 12.69	77.57 ± 13.27	**0.014**	77.66 ± 12.74	77.49 ± 13.28	0.752
Heart rate (beats/min, mean ± SD)	87.98 ± 16.62	87.29 ± 15.87	0.195	88.98 ± 16.02	87.27 ± 15.92	**0.011**
Laboratory tests						
WBC (10^9/L)	7.64 (5.80, 10.32)	7.30 (5.62, 9.75)	**0.006**	7.43 (5.51, 10.22)	7.30 (5.63, 9.82)	0.573
Neutrophil (%) (mean ± SD)	74.92 ± 12.72	72.61 ± 13.02	**<0.001**	74.81 ± 12.61	72.74 ± 12.93	**<0.001**
Hb (g/L) (mean ± SD)	127.14 ± 23.01	126.49 ± 23.36	0.399	128.22 ± 23.28	126.28 ± 23.40	**0.048**
Platelet (109/L) (mean ± SD)	200.74 ± 99.82	204.89 ± 94.84	0.190	193.39 ± 87.63	204.92 ± 95.35	**0.003**
Eosinophils (%) (median, IQR)	0.90 (0.10, 2.60)	1.30 (0.20, 2.90)	**<0.001**	0.95 (0.10, 2.62)	1.30 (0.20, 2.90)	**<0.001**
CRP (mg/L) (median, IQR)	10.90 (3.84, 45.48)	9.37 (3.20, 35.80)	**0.001**	12.70 (5.06, 45.90)	9.66 (3.25, 36.75)	**<0.001**
PCT (ng/ml) (median, IQR)	0.09 (0.05, 0.23)	0.08 (0.04, 0.18)	**0.025**	0.07 (0.04, 0.18)	0.08 (0.05, 0.19)	0.387
ESR (mm/h) (median, IQR)	33.00 (14.00, 61.00)	32.00 (14.00, 60.00)	0.687	36.00 (19.00, 60.00)	32.00 (14.00, 60.00)	0.171
Fibrinogen (g/L) (median, IQR)	3.76 (2.87, 4.98)	3.67 (2.88, 4.82)	0.301	3.59 (2.74, 4.81)	3.69 (2.88, 4.84)	**0.041**
D-dimer (mg/L) (median, IQR)	0.79 (0.41, 1.69)	0.78 (0.39, 1.64)	0.460	0.86 (0.43, 1.75)	0.78 (0.40, 1.70)	0.207
NT-pro-BNP (median, IQR)	342.00 (118.00, 1236.00)	323.55 (86.81, 1455.50)	0.196	295.00 (119.00, 1013.00)	325.50 (88.75, 1464.00)	0.961
PaO2 (mmHg)	88.99 ± 33.95	89.66 ± 31.93	0.579	90.93 ± 35.29	89.64 ± 31.98	0.415
PaCO2 (mmHg)	45.52 ± 12.27	43.39 ± 11.94	**<0.001**	45.57 ± 12.26	43.29 ± 11.83	**<0.001**

AECOPD, acute exacerbation of chronic obstructive pulmonary disease; PSM, propensity score matching; BMI, body mass index; WBC, white blood cell; Hb, hemoglobin; CRP, C-reactive protein; PCT, procalcitonin; ESR, erythrocyte sedimentation rate; NT-proBNP, N-terminal pro-B-type natriuretic peptide; PaO₂, partial pressure of arterial oxygen; PaCO₂, partial pressure of arterial carbon dioxide; OSAHS, obstructive sleep apnea-hypopnea syndrome; IQR, interquartile range; SD, standard deviation. Bold values indicate *p* < 0.05.

## Results

### Study cohort and baseline characteristics

**Figure 1:**
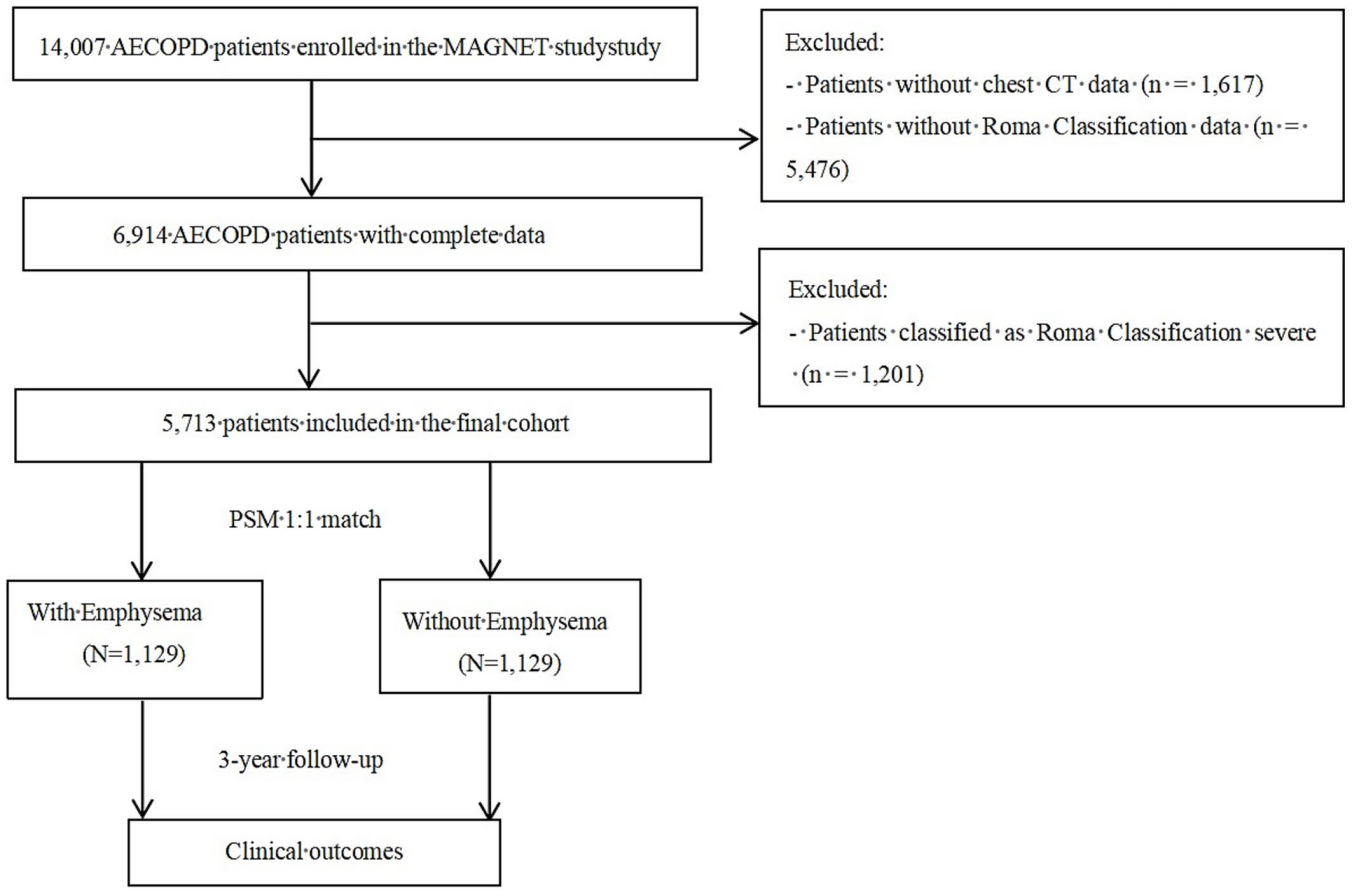
Study flowchart. AECOPD, acute exacerbation of chronic obstructive pulmonary disease; PSM, propensity score matching; CT, computed tomography.

A total of 14,007 patients with AECOPD were initially enrolled from the registry. After excluding 1,617 patients lacking chest CT data and 5,476 patients without Rome severity classification data, 6,914 patients were eligible for further assessment. Subsequent exclusion of 1,201 patients classified with a severe exacerbation by the Rome criteria yielded a final cohort of 5,713 patients for analysis (Figure 1).

Prior to PSM, significant differences were observed between the emphysema (*n* = 4,558) and non-emphysema (*n* = 1,155) groups (Table 1). Patients with emphysema were older (72.63 ± 10.25 vs. 71.01 ± 11.47 years, *p* < 0.001), had a lower body mass index (BMI; 21.43 ± 3.93 vs. 22.81 ± 4.22 kg/m^2^, *p* < 0.001), and included a lower proportion of females (20.7% vs. 31.8%, *p* < 0.001). However, they exhibited higher rates of smoking (19.9% vs. 14.3%, *p* < 0.001) and frequent exacerbations (≥2 times per year; 32.0% vs. 25.7%, *p* = 0.029). No significant differences were observed in the use of long-term oxygen therapy, FEV1/FVC ratio, or percent predicted FEV1 between groups (all *p* > 0.05).

The emphysema group demonstrated significantly lower prevalence rates of hypertension (34.7% vs. 41.0%, *p* < 0.001), heart failure (14.0% vs. 18.4%, *p* < 0.001), obstructive sleep apnea-hypopnea syndrome (OSAHS; 0.4% vs. 1.6%, *p* < 0.001), connective tissue disease (1.4% vs. 2.4%, *p* = 0.020), diabetes (15.1% vs. 17.8%, *p* = 0.025), and osteoporosis (3.3% vs. 4.5%, *p* = 0.049). In contrast, the prevalence of cor pulmonale was significantly higher in the emphysema group (26.6% vs. 19.0%, *p* < 0.001). No significant differences were observed for other comorbidities, including pulmonary hypertension, Parkinson’s disease, chronic renal insufficiency, gastroesophageal reflux, and anxiety or depression (all *p* > 0.05).

According to the Rome classification, the emphysema group comprised a lower proportion of mild cases (48.5% vs. 56.7%) and a higher proportion of moderate cases (51.5% vs. 43.3%, *p* < 0.001). Respiratory failure (10.2% vs. 3.5%, *p* < 0.001), cough (94.3% vs. 91.3%, *p* < 0.001), and purulent sputum production (19.2% vs. 16.6%, *p* = 0.044) were more common in the emphysema group. Additionally, patients with emphysema presented with higher systolic (131.99 ± 19.53 vs. 130.24 ± 19.92 mmHg, *p* = 0.008) and diastolic blood pressures (78.64 ± 12.69 vs. 77.57 ± 13.27 mmHg, *p* = 0.014).

Laboratory analyses revealed consistently elevated inflammatory markers in the emphysema group, including white blood cell count (7.64 [5.80, 10.32] vs. 7.30 [5.62, 9.75], *p* = 0.006), neutrophil percentage (74.92% ± 12.72 vs. 72.61% ± 13.02, *p* < 0.001), C-reactive protein (CRP; 10.90 [3.84, 45.48] vs. 9.37 [3.20, 35.80] mg/L, *p* = 0.001), procalcitonin (PCT; 0.09 [0.05, 0.23] vs. 0.08 [0.04, 0.18] ng/mL, *p* = 0.025), and PaCO₂ (45.52 ± 12.27 vs. 43.39 ± 11.94 mmHg, *p* < 0.001), alongside lower eosinophil counts (0.90 [0.10, 2.60] vs. 1.30 [0.20, 2.90] × 10^9^/L, *p* < 0.001). No significant differences were identified in hemoglobin, platelet count, erythrocyte sedimentation rate (ESR), fibrinogen, d-dimer, N-terminal pro-brain natriuretic peptide (NT-proBNP), or PaO₂ (all *p* > 0.05).

### Propensity score matching

A 1:1 PSM was performed, resulting in a well-balanced cohort of 2,258 patients (emphysema group, *n* = 1,129; non-emphysema group, *n* = 1,129) for subsequent analysis. After matching, the emphysema group maintained a lower BMI (21.57 ± 3.86 vs. 22.82 ± 4.22 kg/m^2^, *p* < 0.001), a higher rate of long-term oxygen therapy (23.3% vs. 14.3%, *p* = 0.037), and a lower frequency of exacerbations (18.8% vs. 26.0%, *p* < 0.001). Gender, age, current smoking status, FEV1/FVC, and percent predicted FEV1 were well-balanced between the matched groups. The distinct comorbidity profile persisted after matching, with the emphysema group showing lower rates of hypertension (32.7% vs. 41.5%, *p* < 0.001), heart failure (13.6% vs. 18.7%, *p* = 0.001), and OSAHS (0.4% vs. 1.6%, *p* = 0.004), but a higher prevalence of cor pulmonale (37.8% vs. 19.0%, *p* < 0.001).

The emphysema group continued to exhibit more severe disease, with a lower proportion of mild cases (46.0% vs. 56.6%) and a higher proportion of moderate cases (54.0% vs. 43.4%) by Rome classification (*p* < 0.001), as well as a higher incidence of pneumonia (33.3% vs. 27.2%, *p* = 0.002) and respiratory failure (12.1% vs. 3.4%, *p* < 0.001). Symptoms including cough (94.5% vs. 91.4%, *p* = 0.005), purulent sputum (32.2% vs. 16.4%, *p* < 0.001), and elevated heart rate (88.98 ± 16.02 vs. 87.27 ± 15.92 bpm, *p* = 0.011) remained more pronounced in the emphysema group. Laboratory findings confirmed persistently higher neutrophil counts (74.81% ± 12.61 vs. 72.74% ± 12.93, *p* < 0.001), CRP levels (12.70 [5.06, 45.90] vs. 9.66 [3.25, 36.75] mg/L, *p* < 0.001), and PaCO₂ (45.57 ± 12.26 vs. 43.29 ± 11.83 mmHg, *p* < 0.001), alongside lower eosinophil counts (0.95 [0.10, 2.62] vs. 1.30 [0.20, 2.90] × 10^9^/L, *p* < 0.001) (Table 1). The balance of matched covariates after PSM is shown in Supplementary Table S1.

### Primary outcome: long-term mortality

**Figure 2:**
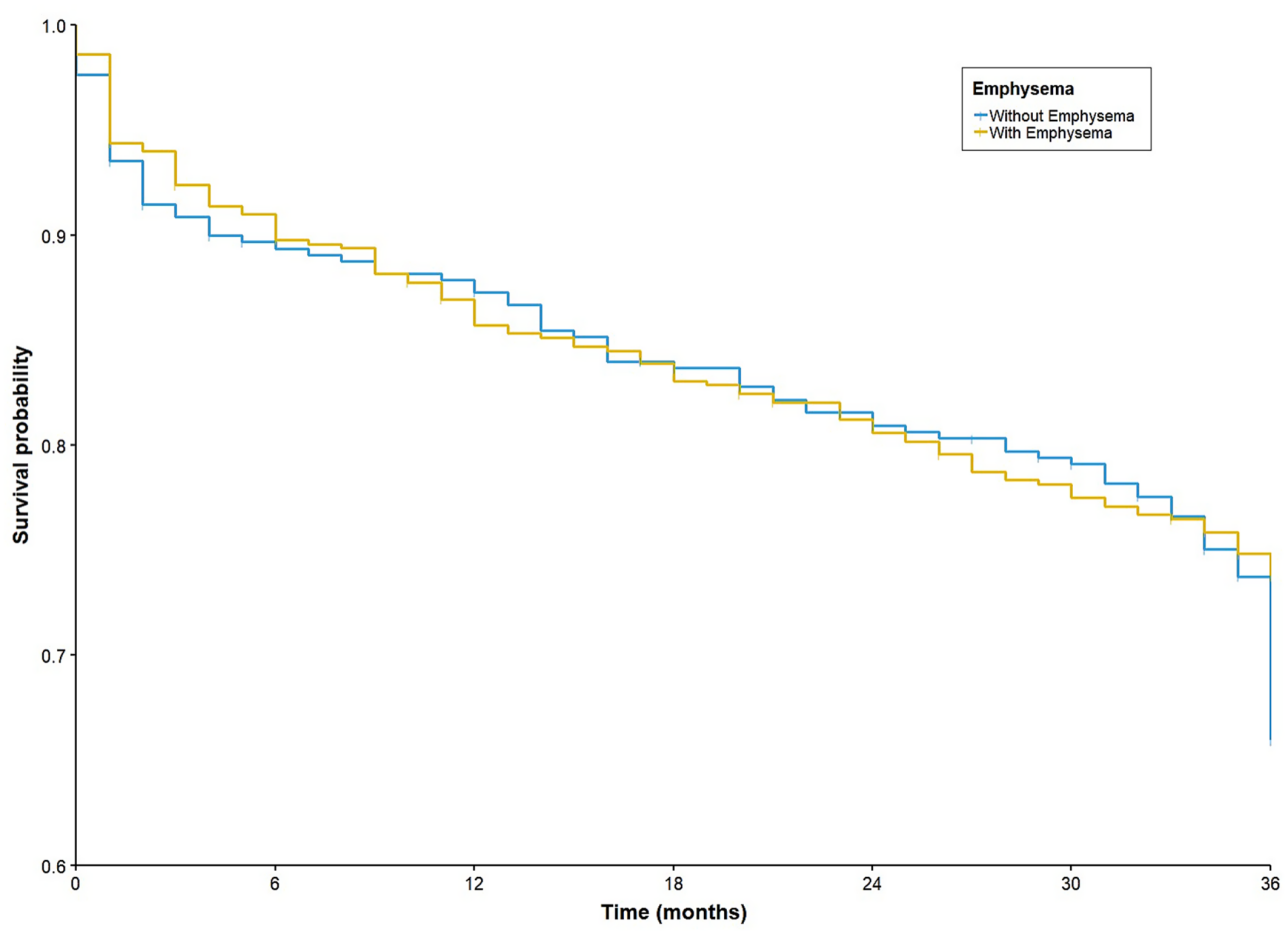
Kaplan–Meier curves for 3-year all-cause mortality in AECOPD patients with and without emphysema. AECOPD, acute exacerbation of chronic obstructive pulmonary disease.

For the analysis of long-term survival, a sub-cohort of 1,115 patients from the West China Hospital was selected from the 2,258 PSM-matched patients. This sub-cohort comprised 645 patients with emphysema and 470 without. A total of 124 patients (19.2%) in the emphysema group and 103 (21.9%) in the non-emphysema group were lost to follow-up. Among them, 521 patients (80.8%) in the emphysema group and 367 (78.1%) in the non-emphysema group completed the 3-year follow-up (Table 2). Kaplan–Meier analysis demonstrated a significantly higher 3-year mortality rate in the non-emphysema group compared to the emphysema group (30.2% vs. 24.8%, log-rank *p* = 0.048; Figure 2). The results of multivariable Cox regression analysis for 3-year all-cause mortality are presented in Supplementary Table S2.

**Table 2:** Comparison of clinical outcomes between AECOPD patients with and without emphysema after propensity score matching.

Variables	With emphysema	Without emphysema	*p*-value
Number of patients	1,129	1,129	/
Short-term outcome			
In-hospital mortality, %	19 (1.7)	25 (2.2)	0.437
Non-invasive ventilation, %	272 (24.1)	216 (19.1)	**0.005**
Invasive ventilation, %	55 (4.9)	51 (4.5)	0.762
ICU admission, %	62 (5.5)	73 (6.5)	0.374
LOS (days, median, IQR)	11.00 (8.00, 15.00)	10.00 (7.00, 14.00)	**<0.001**
Long-term outcome			
Number of patients	645	470	/
Follow-up completed	521	367	/
3-year mortality, %	129/521 (24.8)	111/367 (30.2)	**0.035**
Acute exacerbation readmission, %	251 (50.0)	126 (36.8)	**<0.001**

AECOPD, acute exacerbation of chronic obstructive pulmonary disease; ICU, intensive care unit; LOS, Length of hospital stay; IQR, interquartile range. Bold values indicate *p* < 0.05.

### Comparisons of secondary outcomes

Regarding short-term outcomes, the emphysema group required non-invasive ventilation more frequently (24.1% vs. 19.1%, *p* = 0.005) and had a longer median hospital length of stay (11.00 [8.00, 15.00] vs. 10.00 [7.00, 14.00] days, *p* < 0.001). No significant differences were observed in other in-hospital outcomes, including mortality, invasive ventilation, or ICU admission. During the 3-year follow-up, the emphysema group exhibited a significantly higher rate of readmission due to COPD exacerbation (50.0% vs. 36.8%, *p* < 0.001) (Table 2).

## Discussion

This prospective, multicentre cohort study delineates a distinct clinical profile for the CT-defined emphysema phenotype in patients hospitalized with mild-to-moderate AECOPD. We observed that patients with emphysema presented with more severe acute disease, heightened systemic inflammation, and increased in-hospital healthcare resource utilization. Notably, this group demonstrated a lower risk of 3-year all-cause mortality compared to their non-emphysematous counterparts, although the statistical significance was borderline (*p* = 0.048).

Our finding of a survival advantage in the emphysema phenotype contrasts with previous studies that established emphysema as a predictor of poorer prognosis in broader COPD populations (16, 17). This discrepancy may be explained by distinct drivers of mortality operating at different disease stages. In our cohort, the non-emphysema group, despite less severe respiratory impairment, carried a heavier burden of prognostically significant systemic comorbidities, as evidenced by significantly higher rates of hypertension (41.0% vs. 34.7%) and heart failure (18.4% vs. 14.0%) prior to matching. We hypothesize that the elevated mortality in this group stems from a systemic inflammatory pathophysiology. This phenotype is frequently associated with a “frequent exacerbator” profile, predominantly driven by neutrophilic airway inflammation (18–21). The neutrophil-to-lymphocyte ratio (NLR), a marker of such inflammation, has been independently associated with both all-cause and cardiovascular mortality in COPD (22). Consequently, the pre-existing systemic inflammatory burden is markedly amplified during an acute exacerbation, which could potentially act as a trigger for fatal cardiovascular events. However, this proposed mechanism remains speculative in the absence of cause-specific mortality data.

Conversely, the emphysema phenotype was characterized by a state of acute-on-chronic respiratory decompensation, objectively evidenced by a markedly higher incidence of respiratory failure (10.2% vs. 3.5%) and elevated PaCO₂ (45.52 ± 12.27 vs. 43.39 ± 11.94 mmHg). This respiratory-centric pathophysiology, coupled with a heightened systemic inflammatory state (reflected in higher neutrophil percentage, CRP, and PCT), explains their greater need for acute supportive care such as non-invasive ventilation. We postulate that these patients experience a more indolent progression toward respiratory failure, which is acutely manageable, thereby resulting in the observed “high-morbidity-but-low-mortality” pattern. Collectively, our data suggest that in early-stage AECOPD, the mortality risk conferred by a high comorbidity burden may surpass the direct risk from emphysematous lung destruction itself.

To further elucidate these divergent pathways, our findings can be interpreted through the lens of phenotype-specific pathophysiology. The elevated mortality observed in the non-emphysema group aligns with a profile where systemic comorbidities act as primary determinants of poor outcomes. Prior research confirms that cardiovascular and cerebrovascular comorbidities are significant predictors of mortality in AECOPD patients (23). The physiological stress of an exacerbation, characterized by dynamic hyperinflation and increased metabolic demand, can induce significant cardiac strain, exacerbate underlying pulmonary hypertension, and potentially accelerate progression toward right heart failure (24, 25). Although cause-specific mortality data were not available in our study, it is plausible that this pathophysiological sequence contributes to the higher long-term mortality risk observed in the non-emphysema group, whose prognosis may be governed more by the burden of coexisting cardiometabolic diseases than by primary respiratory failure. However, this interpretation remains speculative, and future studies with detailed cause-of-death data are needed to confirm this hypothesis.

Conversely, the emphysema phenotype in our cohort demonstrated a pathophysiology centered on respiratory system compromise. Foundational research has established a linear relationship between emphysema severity and reductions in cardiac chamber volumes, supporting a mechanism of impaired pulmonary circulation and consequent ventricular underfilling (26–28). This upstream circulatory impairment, evidenced in our data by higher rates of respiratory failure and hypercapnia, delineates a predominantly respiratory form of disability. Crucially, this respiratory-driven trajectory, while responsible for significant morbidity, appears more susceptible to effective long-term supportive management. Robust evidence from randomized controlled trials confirms that non-invasive ventilation significantly reduces mortality and intubation rates in cases of acute hypercapnic respiratory failure (29). Furthermore, the evolving application of long-term home non-invasive ventilation offers a viable strategy for modifying the disease course in chronic hypercapnic respiratory failure, with contemporary studies reporting five-year survival rates exceeding 50 % alongside significant reductions in future exacerbation frequency and hospitalization burden (30). This established management paradigm provides a plausible explanation for the high morbidity but low mortality pattern we observed, as the principal threat faced by the emphysema group, namely respiratory failure, is both treatable in the acute setting and manageable over the long term, thereby mitigating its lethality within our three-year follow-up period.

This study has several notable strengths, including its large sample size derived from a prospective, multicenter registry, which enhances the statistical power and generalizability of our findings. The comprehensive collection of baseline and outcome data, coupled with the application of propensity score matching, strengthens the internal validity of the comparative analyses. Furthermore, to our knowledge, this is the first large-scale study to specifically investigate long-term outcomes in the mild-to-moderate AECOPD phenotype as defined by the Rome classification.

Notwithstanding these strengths, several limitations must be considered. First, the study population was exclusively comprised of hospitalized AECOPD patients; consequently, the findings may not be directly generalizable to individuals with stable COPD or those managed in an outpatient setting. Second, a substantial proportion of patients (49.2%) were excluded, primarily due to incomplete Rome classification data; although chest CT within 24 h is routine in Chinese clinical practice, residual selection bias cannot be completely excluded. Third, and most importantly, the observational nature of this study, despite our methodological adjustments, precludes definitive causal inferences. Residual confounding from unmeasured or imperfectly measured factors (e.g., medication adherence, socioeconomic status, or detailed pharmacological treatments) may persist. Fourth, although the parent MAGNET registry is multicenter in nature, the long-term mortality analysis was conducted using a sub-cohort derived from a single center (West China Hospital). Consequently, the prognostic findings may not be fully representative of the broader multicenter population, and this limitation should be considered when interpreting the generalizability of the 3-year mortality results. The systematic follow-up data from other participating centers are currently being consolidated for future analyses.

Our findings may have implications for clinical practice, suggesting a need to reconsider the conventional “one-size-fits-all” management approach for COPD. For patients presenting with the non-emphysema phenotype, clinical focus might extend beyond the lungs to include screening and management of cardiovascular comorbidities. For those with the emphysema phenotype, optimizing long-term respiratory support could be considered. Future research is needed to elucidate the biological pathways underlying these divergent prognostic trajectories.

## Conclusion

In summary, our study suggests that in mild-to-moderate AECOPD, the emphysema phenotype is associated with higher acute morbidity but lower long-term mortality. This finding indicates a need to revisit the prevailing prognostic model and points toward phenotype-specific drivers of outcomes. These results support further exploration of phenotype-driven management strategies in COPD.

## Data Availability

The raw data supporting the conclusions of this article are not publicly available due to patient privacy restrictions but can be requested from the corresponding author upon reasonable request.
