# Supplementary material for: Clinical and prognostic differences in mild to moderate AECOPD with and without emphysema: a 3-year multicenter prospective study
Source: Front Med (Lausanne). 2026 Jun 24;13:1853642. doi: 10.3389/fmed.2026.1853642 (PMC13341661; doi:10.3389/fmed.2026.1853642)
Supplement: Supplementary file 1 [file Table_1.DOCX]

**Supplementary Table S1. Balance of matched covariates after propensity score matching**

| **Variable** | **Standardized Mean Difference (SMD)** | **Balance Status** |
| --- | --- | --- |
| **Age** | 0.0126 | Balanced (SMD < 0.1) |
| **Sex** | 0.0115 | Balanced (SMD < 0.1) |
| **Current smoking** | 0.0051 | Balanced (SMD < 0.1) |

**Note:** SMD < 0.1 indicates acceptable balance between groups after matching.
