# Supplementary material for: Clinical and prognostic differences in mild to moderate AECOPD with and without emphysema: a 3-year multicenter prospective study
Source: Front Med (Lausanne). 2026 Jun 24;13:1853642. doi: 10.3389/fmed.2026.1853642 (PMC13341661; doi:10.3389/fmed.2026.1853642)
Supplement: Supplementary file 2 [file Table_2.DOCX]

**Supplementary Table S2. Multivariable Cox regression analysis for 3-year all-cause mortality**

| **Variable** | **HR (95% CI)** | **P value** |
| --- | --- | --- |
| Emphysema | 0.77 (0.59-0.99) | 0.047 |
| Age (per 1-year increase) | 1.03 (1.01-1.04) | <0.001 |
| Sex | 0.84 (0.63-1.12) | 0.233 |
| Pneumonia | 1.21 (0.93-1.58) | 0.157 |
| Heart failure | 1.45 (1.06-1.97) | 0.019 |
| Cor pulmonale | 1.50 (1.14-1.96) | 0.003 |
| Diastolic blood pressure (per 1 mmHg increase) | 1.01 (0.99-1.02) | 0.200 |
